# Naringenin accords hepatoprotection from streptozotocin induced diabetes in vivo by modulating mitochondrial dysfunction and apoptotic signaling cascade

**DOI:** 10.1016/j.toxrep.2014.08.002

**Published:** 2014-08-13

**Authors:** Radhika Kapoor, Poonam Kakkar

**Affiliations:** CSIR-Indian Institute of Toxicology Research, M.G. Marg, Lucknow 226001, India

**Keywords:** ΨΔm, mitochondrial membrane potential, AGE, advanced glycated end products, AIF, apoptosis inducing factor, Bax, bcl-2 associated X, Bcl-2, b-cell Lymhoma-2, CAT, catalase, cDNA, complementary, DNA,COX-II, cyclo-oxygenase-II, DCF, dichlorofluorescein, DCFH-DA, 2′7′dichlorofluorescein diacetate, EDTA, ethylenediaminetetraacetic acid, Endo-G, endonuclease-G, FITC, fluorescein isothiocyanate, GAPDH, glyceraldehyde 3 phosphate dehydrogenase, GPx, glutathione peroxidase, GSH, reduced glutathione, HRP, horseradish peroxidase, JC-1, 5,5′,6,6′-tetrachloro-1,1′,3,3′-tetraethylbenzimidazol-carbocyanine iodide, MPT, mitochondrial permeability transition, NADPH, nicotinamide adenine dinucleotide phosphate reduced, NBT, nitroblue tetrazolium, p-NA, p-nitro aniline, PBS, phosphate buffered saline, PKC, protein kinase-C, PVDF, polyvinylidene difluoride, ROS, reactive oxygen species, RT-PCR, reverse transciptase polymerase chain reaction, SOD, superoxide dismutase, Ctrl, control rats, Diab, diabetic rats, CoN, diabetic rats co-treated with naringenin during streptozotocin induction, PoN, diabetic rats treated with naringenin after diabetes induction, Glib, diabetic rats treated with standard drug glybenclamide, Sil, diabetic rats treated with silymarin, CtN, control rats treated with naringenin, Naringenin (PubChem CID: 932), Streptozotocin (PubChem CID: 29327), Nicotinamide (PubChem CID: 936), d-glucose (PubChem CID: 5793), Silymarin (PubChem CID: 1548894), Glibenclamide (PubChem CID: 3488), NADPH (PubChem CID: 12598259), Thiobarbituric acid (PubChem CID: 3081198), TriChloroacetic acid (PubChem CID: 6421), Sodium dodecyl sulphate (PubChem CID: 3423265), Apoptosis, Streptozotocin induced diabetes, Mitochondrial dysfunction, Oxidative stress, Naringenin, Liver damage

## Abstract

Diabetic complications cause noticeable liver damage, which finally progresses to diabetic hepatopathy. Nutritive antioxidants not only reduce the liver damage, but also prevent it by modulating the release of various proteins involved in apoptotic signaling cascades. This study explores the molecular mechanisms underlying diabetes-induced liver damage and its modulation by naringenin. Antioxidant status, liver & kidney biomarker enzymes, reactive oxygen species (ROS) generation, mitochondrial membrane potential, expression of apoptotic proteins like Bax (bcl-2 associated X), Bcl-2 (b-cell Lymhoma-2), Caspase-3, Caspase-9, AIF (Apoptosis inducing factor) and Endo-G (Endonuclease-G) were studied in streptozotocin induced diabetic rats. Significant hyperglycemia, disturbed antioxidant status, altered carbohydrate metabolizing enzymes, increased ROS and lipid peroxidation; decreased mitochondrial membrane potential and enhanced release of AIF and Endo-G were observed. Hyperglycemia also affected apoptosis and its related genes at both transcriptional and translational level (Caspase-3 & 9, Bax and Bcl-2) in the liver of diabetic rats. Naringenin, a flavonone, exerted anti-hyperglycemic effect and was able to prevent oxidative stress and resultant apoptotic events caused due to diabetes-induced hepatotoxicity. Thus, our study shows, a protective effect of naringenin against diabetes induced liver damage and redox imbalance, which could further be exploited for the management of diabetic hepatopathy.

## Highlights

• Streptozotocin induced diabetes altered fasting blood glucose, body weight and carbohydrate metabolizing enzymes.
• Diabetes induced ROS, enhanced lipid peroxidation and protein carbonyl content while down-regulating antioxidants.
• Apoptotic proteins like AIF, Endo-G, Bax, Bcl-2, caspase-3 & 9 were significantly modulated during diabetes.
• Naringenin modulated the fasting glucose, carbohydrate metabolizing enzymes and antioxidant status of diabetic rats.
• Naringenin inhibited mitochondrial depolarization and up/down regulation of apoptotic/antiapoptotic proteins.

## 1 Introduction

Diabetes mellitus, characterized by high blood sugar, is a disorder related to deficiency of insulin secretion/action or both. The prevalence and mortality due to this chronic metabolic disease have been reported in both developed and developing countries. Though the leading mechanism of diabetic complications remains unclear, oxidative damage is considered as one of the major causative factors [12], [32], [33]. Scientific reports show enhanced generation of reactive oxygen species (ROS) in both types of diabetes (Type-1 and Type-2), which relates oxidative stress to the onset of diabetes [10]. Four important metabolic pathways namely PKC (Protein Kinase-C), hexosamine, advanced glycation and polyol pathway induce overproduction of reactive oxygen species (ROS) in mitochondria during hyperglycemia [6]. This excessive ROS generation, in turn, induces various metabolic dysfunctions and contributes to progressive development of micro-/macro vascular complications and multi-organ damage.

Liver is the main organ of glucose homeostasis, oxidative processes and detoxification site for major metabolites produced as a repercussion of excessive ROS and holds importance during the progression of diabetes. Hence, the inhibition of ROS generation can be an important strategy to restore normal functioning of the liver and control the progression of diabetes-induced liver damage. Apoptosis is a common form of cell death morphologically characterized by cellular shrinkage, chromatin condensation, DNA fragmentation and cell disruption into apoptotic bodies. Generation of excessive free radicals due to hyperglycemia and onset of apoptosis has been reported in scientific literature [35], [42]. Release of apoptotic proteins like AIF (Apoptosis Inducing factor), Endo-G (Endo nuclease-G), Bax (bcl-2 associated X), Bcl-2 (b-cell Lymhoma-2) and caspases has been well demonstrated by our group during drug induced hepatotoxicity and hyperglycemia under in vitro conditions [14], [15], [34].

Various researchers suggest that phytochemical from traditionally known medicinal plants have been extensively used as an alternative medicine for the management of diabetes. Literature suggests that flavanones are potentially used as an anti-diabetic agent due to their chemical structure [23]. Recent scientific reports show that naringenin suppresses carbohydrate absorption in the intestine and thereby prevents the increased post-prandial glucose in diabetic patients [5]. A recent study showed naringenin to be better as compared to hesperedin and hesperitin (its related citrus flavanones) as far as interaction with intracellular enzymes, mitochondrial and cellular membrane is concerned. Moreover, the inhibition of the gluconeogenic pathway by the citrus flavanones like naringenin has been found similar to that of metformin in a recent report [5]. We initially studied the effect of several flavanones against high glucose induced cell death in hepatocytes (data not shown) out of which naringenin was selected for the present study. Flavonoids are widely recognized as capable of lowering both ROS generation and high circulating blood glucose. Naringenin, a flavanone, is considered to have beneficial effects on human health. It is considered a good anti-inflammatory agent, improves carbohydrate metabolism and immune system. It is one of the major constituents of grapefruit, *Citrus paradisi*, which is traditionally known to have medicinal importance. We have previously reported that naringenin exerts its protective effect in high glucose (40 mM) stressed hepatocytes in vitro by inhibiting the release of apoptotic proteins and maintaining antioxidant status of the cells [15]. Recent research reports suggest the anti hyperglycemic effect of naringenin [3], [29], but to the best of our knowledge, mechanism involved in apoptosis caused during diabetes induced liver damage and its amelioration by naringenin has not been reported.

In view of the above findings, the present study aims to unveil the anti hyperglycemic potential of naringenin by exploring its protective action against apoptosis in streptozotocin induced diabetic rats in vivo. The results obtained from the present study suggest that naringenin prevents hyperglycemia-induced apoptotic cell death by modulating ROS generation and mitochondria mediated apoptotic cell death. Besides acting as an antioxidant, naringenin has an important preventive role in hyperglycemia-induced liver damage.

## 2 Materials and methods

### 2.1 Chemicals

All chemicals used in the study were procured from Sigma Chemicals Co. (St. Louis, MO, USA). Chemicals used from other sources have been stated otherwise.

### 2.2 Animals

Male Wistar rats of 150–200 g from Indian Institute of Toxicology Research (IITR) animal colony were used in the study. Rats were kept under standard condition of 25 ± 2 °C temperature, 60–70% humidity and a controlled 12 h light/dark cycle. Rats were given a standard pellet diet (Ashirwad Pellet Diet, Mumbai, India) and water ad libitum. Animal handling in all experimental procedures was approved by the Institutional Animal Ethics Committee, (ITRC/IAEC/22/2011). The animals were randomized into control and experimental groups and housed in different cages.

### 2.3 Induction and assessment of diabetes mellitus

In Wistar rats, diabetes was induced by single intraperitoneal injection of streptozotocin (65 mg/kg bwt) dissolved in normal saline. Prior to diabetes induction, rats were treated with 110 mg/kg bwt. nicotinamide. Nicotinamide is used prior to streptozotocin injection so that the beta-cytotoxic effect of streptozotocin is partially protected. Furthermore, when compared to available diabetic models, the diabetic rat model developed with this protocol is found closer to non-insulin-dependent diabetes mellitus with regard to insulin responsiveness to glucose and sulfonylureas [27]. After four days of streptozotocin treatment, rats were fasted overnight and identified as diabetic on the basis of blood glucose levels (higher than 250 mg/dL) in tail blood using Glucometer (MediSense Optimum TM Xceed, Abott Diabetes Care Inc. Almeda, USA).

#### 2.3.1 Oral glucose tolerance test

For oral glucose tolerance test (OGTT), five control rats were grouped in one group while 25 diabetic rats were further divided into five groups (*n* = 5) from Group II to Group VI. After 18 h fasting, initial blood-glucose level (BGL) was estimated (0 min). Glucose solution (2 g/kg bwt) was administered by oral gavage from group I to group VI. At the same time, group III, IV and V received naringenin at a dose of 25 mg/kg bwt, 50 mg/kg bwt and 100 mg/kg bwt orally, respectively, while group VI received standard drug glybenclamide (0.6 mg/kg bwt). Blood glucose was further monitored at four more cardinal points at time intervals of readings 30, 60, 90, and 120 min after glucose and test sample administration. Set of animals used in this experiment is different from that of used in the main study; the aim of this particular experiment was to find out an effective dose of naringenin against hyperglycemia induced apoptosis in liver of rats. 100 mg/kg bwt dose of naringenin is refereed as the human relevant dose in the literature [39]. Study of different doses of naringenin lower than 100 mg/kg bwt, can give insight into the possible human use of lower but, effective dose.

#### 2.3.2 Treatment schedule

On the basis of oral glucose tolerance test, effective dose of naringenin (50 mg/kg bwt) was selected for further experiments. Diabetic rats and control rats were further divided into groups of six animals per group. Dose regimen was started from the next day after confirmation of non-insulin-dependent diabetes mellitus and continued up to 30 days. Detail of experimental groups is as under:

**Table:** 

Group 1	Control rats given normal saline; **Ctrl**
Group 2	Diabetic rats given normal saline; **Diab**
Group 3	50 mg/kg Naringenin given along with diabetes induction; **Co-naringenin**; **CoN**
Group 4	50 mg/kg Naringenin given to diabetic rats; **Post-naringenin; PoN**
Group 5	Diabetic rats given Glibenclamide 0.6 mg/kg (positive drug control); **Glib**
Group 6	Diabetic rats given Silymarin 200 mg/kg; **Sil** (positive phytochemical control)
Group 7	Control rats given Naringenin 50 mg/kg; **CtN**

In one of the previous studies from our lab [37], silymarin administered to rats at 200 mg/kg bwt did not exhibit any toxicity or significant change in antioxidant enzymes, hence, to save animals, group of control silymarin was not included in the study.

### 2.4 Measurement of general clinical parameters

#### 2.4.1 Measurement of body weight and fasting blood glucose

The body weight of all groups of animals was monitored daily at a fixed time. Fixed amount of rat food and fluid was given to each group and refilled the next day. Fasting blood glucose was regularly monitored weekly.

#### 2.4.2 Liver function tests through biochemical parameter analysis

Serum was separated from the blood. The activities of glutamic oxaloacetic transaminase (GOT), Glutamic Pyruvic Transaminase (GPT), ALP (alkaline phosphatase), cholesterol, blood glucose, level of creatinine, albumin, total bilirubin, blood urea nitrogen, and total protein were measured using clinical autoanalyzer (Chemwell 2910, USA).

#### 2.4.3 Tissue collection and preparation of homogenate

All animals were euthanized using chloroform and sacrificed. Blood was collected from the jugular vein of animals in EDTA coated vials. Tissue was collected in ice cold phosphate buffer saline (PBS), weighed and homogenized. Tissue homogenate (10% w/v) was made in 10 mM sodium phosphate buffer using an electric motor with Teflon glass and pestle [20]. The entire procedure was carried out under cold conditions.

### 2.5 Protein estimation

Protein estimation was done by using standard protocol of [21]. Bovine serum albumin was used as standard and the color developed was read at 660 nm on Spectramax Plus 384 (Molecular Devices, USA).

### 2.6 Carbohydrate metabolizing enzyme assays

#### 2.6.1 Glucokinase

For Glucokinase activity (EC: 2.7.1.2), 100 μl of 10% tissue homogenate was added to the reaction mixture containing 0.1 M Tris buffer (pH = 7.4), 0.2 mm NADP^+^, 5 mM ATP, 5 mM MgCl_2_, 0.3 units of glucose-6-phosphate dehydrogenase, 37 mM glucose [28]. Increase in absorbance was measured for 3 min at intervals of 30 s at 340 nm. Glucokinase activity is expressed as μM NADPH (Nicotinamide adenine dinucleotide phosphate reduced), decomposed/min/mg protein.

#### 2.6.2 Glucose-6-phosphate dehydrogenase

The liver glucose-6-phosphate dehydrogenase (EC: 1.1.1.49) activity was measured at 340 nm in a reaction mixture containing 0.2 M Tris–HCl buffer (pH = 7.5), 0.5 × 10^−2^ M glucose-6-phosphate, 2 × 10^−4^ M NADP and 0.04 M MgCl_2_ [17]. Increase in absorbance was measured for 3 min at 30 s intervals. Glucose-6-phosphate dehydrogenase activity is expressed as μM NADPH decomposed/min/mg protein.

### 2.7 Assessment of oxidative stress

#### 2.7.1 Determination of thiobarbituric acid-reactive substances

Level of lipid peroxidation was measured by the method of [40]. Malondialdehyde (MDA) reacts with thiobarbituric acid (TBA) to yield a colored compound. Briefly, 10 μl homogenate was added to 70 μl of double distilled water, 50 μl of 50 mM phosphate buffer, 10 μl of 1 mM butylated hydroxyl toluene, 75 μl of 1.3% TBA. 50 μl of 50% trichloroacetic acid was added to the reaction. The mixture was then incubated at 60 °C for 40 min and kept on ice for 15 min. The reaction was stopped by the addition 10 μl of 20% SDS (Sodium dodecyl sulfate). Absorbance was measured at 530 nm and 600 nm and results expressed as nM MDA formed/μg protein using freshly diluted 1,1,3,3-tetraethoxypropane to draw a standard curve.

#### 2.7.2 Superoxide dismutase (SOD) activity

SOD activity in tissues of all the rats was estimated by using the method of [11]. Briefly, 10 μl of homogenate, 90 μl of 30 mM sodium tetra pyrophosphate buffer (pH 8.3), 30 μl of 0.3 mM Nitro blue tetrazolium (NBT), 10 μl of 0.96 mM phenyl methyl sulphonate (PMS) and 40 μl of DDW was added. The reaction was initiated by the addition of 20 μl 0.72 mM NADH. The reaction was stopped by adding 50 μl of glacial acetic acid. Absorbance was measured at 560 nm. A single unit of enzyme is expressed as 50% inhibition of NBT (Nitro Blue Tetrazolium) reduction/min/mg protein. Results were expressed as a unit of SOD/min/mg protein.

#### 2.7.3 Catalase activity

Catalase activity was assayed spectrophotometrically using the method of [2]. Briefly, the assay mixture of 1.5 ml contained 980 μl of 50 mM sodium phosphate buffer pH 7.0 and 20 μl of homogenate (10–15 μg protein). The reaction was started by addition of 500 μl of 30 mM hydrogen peroxide. The decrease in absorbance was observed for 1 min at every 15 s at 240 nm. Catalase activity is expressed as μmole H_2_O_2_ decomposed/min/mg protein.

#### 2.7.4 Glutathione peroxidase

Glutathione peroxidase (GPx) activity was measured by using the method of [24]. The activity was expressed as nmol NADPH oxidized/min/mg protein.

#### 2.7.5 Reduced glutathione and glutathione disulfide (GSH & GSSG)

GSH and GSSG were measured using the method described by [7] with modifications. 50 μl of tissue homogenate was diluted with 50 μl of 100 mM phosphate buffer containing 1 mM EDTA (Ethylenediaminetetraacetic acid). To this mixture 100 μl of reaction buffer (295 μM 5, 5′-dithio-bis (2-nitrobenzoic acid) (DTNB) was added and estimated at 412 nm immediately. The reduced GSH standard was employed to obtain a standard curve. Reduced GSH is expressed as μM GSH/mg tissue. For estimation of GSSG, 5 μM NADPH was added to the reaction mixture. GSSG was expressed as μM GSSG/mg tissue.

### 2.8 Protein carbonyl content

Reactive free radical oxidizes proteins and leads to structural and functional changes. Protein carbonyl content was estimated by the method of [19]. The homogenate was diluted in a ratio 1:40 with phosphate-buffered saline (PBS) and centrifuged twice for 5–10 min at 14,000 × *g*. 20% TCA chilled was used to precipitate the diluted proteins followed by centrifugation for 3–5 min. A solution of 10 mM DNPH in 2 N HCl was added to the protein pellet of each sample. Samples were allowed to stand in the dark at room temperature for 1 h with vortexing every 10 mins; and further precipitated with 10–20% TCA (final concentration) and centrifuged for 5 min. The supernatants were discarded; the protein pellets were washed once more with 10–20% TCA, and then washed three times with 1 ml of ethanol/ethyl acetate (1:1, v/v) to remove any free DNPH. Samples were then resuspended in 6 M guanidine hydrochloride (GdmCl, dissolved in 2 N HCl) at 37 °C for 15 min with vortex mixing. Carbonyl content was determined from the absorbance at 366 nm using a molar absorption coefficient of 22,000 M^−1^ cm^−1^and expressed as nanomoles per milligram of protein.

### 2.9 Measurement of ROS production in treated rats

ROS generation in rats of all groups was measured by the method of [4] using Varioskan fluorescent microplate reader. The method employs the measurement of ROS generation using the cell permeable fluorescent dye DCFH-DA (2′, 7-dichlorofluoresceindiacetate). Upon entering the cell, DCFH-DA is cleaved by the intracellular esterases leaving DCFH (dichlorofluorescein) which is oxidized to DCF by the oxidants and its fluorescence is taken as an indicator of oxidant production in the cell. In brief, hepatocytes of different treatment groups were isolated and incubated for 30 min with DCFH-DA (5 μg/ml) at 37 °C in the dark.

### 2.10 Mitochondrial membrane potential in treated rats

The effect of the test substance on mitochondrial membrane potential was assessed using JC-1 (5,5′,6,6′-tetrachloro-1,1′, 3,3′-tetraethylbenzimidazol-carbocyanine iodide) flouroprobe. Hepatocytes isolated from rats of different groups were incubated with 2.5 μg/ml of JC-1 flouroprobe at 37 °C in the dark for 30 min. ΔΨm was assessed by comparing the fluorescence (red) (Ex/Em-580/590 nm)/green (Ex/Em-510/527 nm) using Varioskan fluorescent microplate reader [14].

### 2.11 Expression of apoptotic and antiapoptotic genes

Liver tissue samples were collected on ice after sacrifice and processed immediately for RNA isolation. Total RNA was isolated from rats of treated and control groups by using the Trizol reagent (Invitrogen, Carlsbad, CA, USA) according to manufacturer's instructions. 3 μg of total RNA was reverse transcribed (RT) into cDNA using Revert Aid H minus First Strand cDNA Synthesis Kit (Fermentas, EU) as per manufacturer's instructions. Complementary DNA was used for semi-quantitative PCR (polymerase chain reaction) using sets of specific primers as shown in Table 1. cDNA amplification was carried out according to the respective temperature profile and the number of cycles for Bax, Bcl-2, Caspase3 and Caspase 9. GAPDH (Glyceraldehyde 3-phosphate dehydrogenase) was used as internal control. The product was run on a 1.5% agarose gel and photographed on Ultraviolet transilluminator. Image J software 1.44 (USA) was used to analyze PCR products. The result obtained was normalized to GAPDH.

**Table 1:** Primers used for expression of apoptotic and anti-apoptotic genes.

Genes	Primers
Bcl-2	F 5′-ACTTTGCAGAGATGTCCAGTCAG-3′ R 5′-GTTCAGGTACTCAGTCATCCACAG-3′

Bax	F 5′-GGAGGAAGTCCAGTGTCCAG-3′ R 5′-TGCAGAGGATGATTGCTGAC-3′

Caspase-3	F 5′-GAACGAACGGACCTGTGGACCT-3′ R 5′-GCCTCCACTGGTATCTTCTGGCAT-3′

Caspase-9	F 5′-TGAGCCAGATGCTGTCCCATACCAG-3′ R 5′-CCTGGGAAGGTGGAGTAGGACAC-3′

GAPDH	F 5′-GGCCAAGATCATCCATGACAACT-3 R 5′-ACCAGGACATGAGCTTGACAAAGT-3′

### 2.12 Sub-cellular fractionation

Liver nuclear extracts were obtained as described by [18]. Briefly, tissue was washed with PBS, homogenized and suspended in lysis buffer containing 10 mM HEPES, pH 7.9, 10 mM KCl, 0.1 mM EDTA, supplemented with 1 mM dithiothreitol (DTT), protease inhibitor cocktail procured from Sigma Chemicals Co. (St. Louis, MO, USA) and 0.4% NP-40. After 30 min on ice, the lysate wase centrifuged (Sigma 3K18, Germany) at 16,000 × *g* in a micro centrifuge for 5 min at 4 °C. The supernatant was discarded, and the pellet (nuclear fraction) was re-suspended in nuclear extract buffer (20 mM HEPES, pH 7.9, 0.4 mM NaCl, 1 mM EDTA, supplemented with 1 mM DTT, a complete mini protease inhibitor cocktail tablet and 10% glycerol). Pellet samples with extraction buffer were incubated at 4 °C for an hour. The nuclear fraction was sonicated (Sartorius, Lab sonic MTM) for 10 s, and centrifuged at 16,000 × *g* at 4 °C for 5 min. The supernatant was then collected and used as a nuclear extract.

Cytosolic and mitochondrial fractions were prepared as described by [36]. Briefly, tissue homogenates were prepared in ice-cold RIPA buffer. The homogenate was suspended in ice-cold RIPA buffer (20 mM HEPES-KOH, pH- 7.5, 10 mM KCl, 1.5 mM MgCl_2_, 1.0 mM dithiothreitol, 1.0 mM EDTA, 1.0 mM EGTA, 1.0 mM phenylmethylsulfonyl fluoride) and sonicated for 10 s (Sartorius, Lab sonic MTM). The homogenate was centrifuged (Sigma 3K18, Germany) at 800 × *g* for 4 min at 4 °C. Supernatant was then centrifuged at 22,000 × *g* for 15 min at 4 °C. Resulting supernatant was used as cytosolic fraction and pellet (mitochondria) was re-suspended in cold RIPA buffer, incubated on ice for 30 min followed by sonication for 10 s. The homogenate was centrifuged at 15,000 × *g* for 15 min at 4 °C. The resulting supernatant was mitochondrial fraction.

### 2.13 Immunoblot analysis

The protein content corresponding to each treatment was quantified using the method of [21]). Samples were incubated with loading dye for 5 min at 96 °C and immediately kept on ice. Sixty microgram of protein sample from cytosolic or mitochondrial fraction was separated on 12% SDS – polyacrylamide gel electrophoresis and electro blotted on polyvinylidene difluoride (PVDF) membrane (Hybond™ P Amersham biosciences, UK limited, NA). The membrane was blocked for non-specific sites with 1X blocking buffer and washed using Tris buffered saline (TBS) containing 0.1% Tween-20. The membrane was then incubated for 1 h with specific polyclonal IgG antibodies of AIF, Endo-G, Cox IV, Lamin-B and β-Actin (Santa Cruz Biotechnology, Inc.) at 1:500 dilutions. This was followed by washing with PBS and incubated with respective secondary antibody in 1:1000 dilutions. The membrane was processed and developed using ImmobilonTM Western Chemiluminescent Horse Raddish peroxidase substrate kit. (Millipore, Corporation, MA, USA). β-actin was used as internal standard. PageRulerTM Prestained Protein Ladder (SM-0671 from Fermentas, EU) was used to determine the molecular weight of the protein bands. Densitometry of the bands obtained was done using NIH software Image J version 1.41 (USA). The result is expressed as arbitrary units for each experimental band.

### 2.14 Asp-Glu-Val-Asp-Specific caspase activity

Caspase activity was assayed by reaction in the isolation buffer containing 10 mM Tris–HCl buffer, pH 7.6, 5 mM MgCl_2_, 1.5 mM potassium acetate, 2 mM DTT (Dithiothreitol) and protease inhibitor cocktail. To the reaction mixture was added 50 μg of cytosolic protein and 50 μM Asp-Glu-Val-Asp-pNA in the isolation buffer. General caspase activity was evaluated by enzymatic cleavage of chromophore *p*-nitroanilide (PNA) from the substrate *N*-acetyl-Asp-Glu-Val-Asp-pNA (Sigma). The reaction mixtures were incubated at 37 °C for 1 h, and the formation of pNA was measured at 405 nm using Spectramax PLUS 384 microplate reader (Soft max pro version 5.1; Molecular Devices, USA) [31].

### 2.15 Statistical analysis

Data are expressed as mean ± SE. Data was analyzed on SPSS software version 14.0 using student's *t*-test and one-way ANOVA (Analysis of variance). *P < 0.05, **P < 0.01, ***P < 0.001 were used as the criterion for significance.

## 3 Results

### 3.1 Oral glucose tolerance test, fasting blood glucose and body weight

**Fig. 1:**
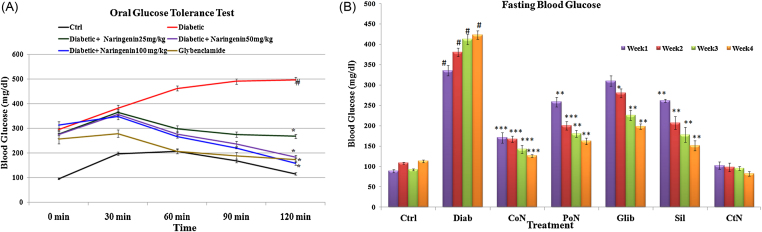
Dose optimization of naringenin and the effect of naringenin on fasting blood glucose. (a) Effect of three doses of Naringenin, i.e., 25 mg/kg bwt, 50 mg/kg bwt and 100 mg/kg bwt on blood glucose during oral glucose tolerance test. (b) Fasting blood glucose in control, diabetic and treated diabetic rats. Each value represents the mean ± SE of six rats. # Denotes significant difference compared with control rats. *P < 0.05, **P < 0.01 and ***P < 0.001 denotes significant difference compared with diabetic control.

After the induction and confirmation of the diabetic condition, the animals were subjected to oral glucose tolerance test (OGTT). Fig. 1 shows the glycemic profile assessed in rats 30, 60, 90 and 120 min after the administration of glucose. A constant increase in blood glucose was observed in the diabetic group until 90 min after administration of glucose, which subsequently declined at 120 min. However, the pattern was not observed in the untreated diabetic group, which continued to have elevated blood glucose at the culmination of the test. Blood glucose reached its peak around 30 min after glucose administration and maximum reduction in blood glucose was observed at 90 min in glybenclamide as well as naringenin treated rats. A dose of 50 mg/kg bwt and 100 mg/kg bwt of naringenin reduced blood glucose by 63% and 68% respectively, in 120 min and the response was comparable to standard drug glybenclamide. Since 50 mg/kg bwt and 100 mg/kg bwt naringenin showed an almost similar response, a lower dose of naringenin was taken up for further experiments (Fig. 1).

Though the initial weight of all groups was almost similar, induction of diabetes caused a significant reduction (*P* < 0.001) in body weight. Naringenin, during its co-treatment, prevented the decrease in body weight caused due to diabetes. A significant increase of 2.08-fold in body weight of diabetic rats was observed when co-treated with naringenin as compared to untreated diabetic rats. However, with respect to control, no significant change was observed in weight of control rats treated with naringenin alone (supplementary data Fig. S1).

A significant (*P* < 0.001) increase of 3.48-fold in fasting blood glucose of diabetic rats was observed when compared to control rats and the increase was up to 4.12-fold at the end of four weeks. It was observed, when naringenin (50 mg/kg bwt) was given prior to the injection of streptozotocin (co-treatment of naringenin), it prevented the increase of blood glucose in diabetic range of 250 mg/dl. However, non-significant increase in blood glucose due to streptozotocin was observed, which was restored to normal levels by further three-week exposure of naringenin. Diabetic rats treated with naringenin (50 mg/kg bwt) showed significant decline of 70.01% (*P* < 0.001) in fasting blood glucose level. However, standard drug glybenclamide, decreased the fasting blood glucuose level by 53.10% as compared to untreated diabetic rats. Control rats treated with naringenin did not show any significant change in fasting blood glucose level (Fig. 1).

### 3.2 Biochemical parameters

A significant (*P* < 0.001) elevation in the activity of marker hepatocyte enzymes, GOT (3.16-fold) and GPT (2.22-fold); renal marker creatinine (2.85-fold), ALP (5.73-fold) and total cholesterol (2.02-fold) (Table 2) in serum of diabetic animals was observed. Naringenin significantly (*P* < 0.001) modulated the levels of liver and kidney marker enzymes, cholesterol and glucose levels. Naringenin (50 mg/kg bwt) was found effective during both the treatment schedules (co and post) which were comparable to standard drugs, glybenclamide and known antioxidant silymarin. However, no change was observed in control rats treated with naringenin alone.

**Table 2:** Naringenin modulated the serum biochemical parameters in rats altered due to induced diabetes.

	SGOT (U/L)	SGPT (U/L)	ALP (U/L)	Creatinine (mg/dl)	Total glucose (mg/dl)	Cholesterol (mg/d l)
Ctrl	85.9 ± 5.8	234.7 ± 9.4	66.5 ± 7.9	0.5 ± 0.01	76.7 ± 4.9	122.4 ± 11.2
Diab	269.3 ± 7.3^#^	599.4 ± 15.6^#^	381.3 ± 11.0^#^	1.2 ± 0.05^#^	494.8 ± 7.5^#^	255.9 ± 13.4^#^
CoN	114.8 ± 8.5^***^	245.8 ± 11.1^***^	64.5 ± 3.9^***^	0.5 ± 0.02^***^	94.8 ± 5.2^***^	119.7 ± 8.8^***^
PoN	127.9 ± 5.8^***^	270.9 ± 12.8^***^	74.32 ± 5.4^***^	0.5 ± 0.03^***^	128.8 ± 6.4^***^	118.9 ± 0.7^***^
Glib	150.3 ± 8.4^**^	289.5 ± 14.6^***^	130.9 ± 8.6^**^	0.6 ± 0.05^**^	203.8 ± 16.5^*^	122.2 ± 1.2^**^
Sil	132.4 ± 10.7^**^	254.8 ± 11.1^**^	102.5 ± 4.4^***^	0.4 ± 0.01^**^	144.5 ± 10.3^**^	132.6 ± 8.6^**^
CtN	75.6 ± 5.9	241.9 ± 19.6	61.7 ± 8.3	0.4 ± 0.02	77.2 ± 8.1	91.2 ± 2.8

Each value represents the mean ± SE of six rats. Ctrl: Control rats; Diab: Diabetic rats; CoN: Diabetic rats co-treated with naringenin during streptozotocin induction; PoN: Diabetic rats treated with naringenin after diabetes induction; Glib: Diabetic rats treated with standard drug glybenclamide; Sil: Diabetic rats treated with Silymarin; CtN: Control rats treated with naringenin. ^#^ Denotes significant difference compared with control rats; ^*^P < 0.05; ^**^P < 0.01; ^***^P < 0.001 denotes significant difference compared with diabetic control.

### 3.3 Antioxidant status of diabetic rats

#### 3.3.1 Catalase activity

Catalase activity was significantly (*P* < 0.001) reduced by 69.07% in the liver of diabetic rats as compared to non-diabetic rats. Diabetic rats co- and post- treated with naringenin exhibited significantly higher catalase activity compared to untreated diabetic rats. Significant enhancement of 2.24-fold (*P* < 0.001; co-treatment) and 2.67-fold (*P* < 0.001; post-treatment) in catalase activity was observed in naringenin (50 mg/kg bwt) treated animals (Table 3). No significant change in the activity of catalase was observed in control rats treated with naringenin.

**Table 3:** Effect of naringenin on antioxidant status of diabetic rats.

Groups/activity	SOD activity (SOD units/min/mg protein)	Catalase activity (μmole H_2_O_2_ decomposed/min/mg protein)	GPx activity (nmol NADPH oxidized/min/mg protein)	Redox ratio (GSH/GSSG)
Ctrl	55.4 ± 1.5	12.2 ± 0.4	102.1 ± 3.3	4.8 ± 0.2
Diab	23.6 ± 1.8^#^	3.7 ± 0.6^#^	48.2 ± 2.8^#^	0.9 ± 0.1^#^
Con	45.5 ± 1.7^***^	8.3 ± 0.9^**^	75.4 ± 2.4^**^	3.4 ± 0.6^**^
PoN	47.5 ± 1.5^**^	9.9 ± 0.8^**^	85.7 ± 2.2^**^	3.4 ± 0.5^***^
Glib	40.0 ± 1.4^**^	7.0 ± 0.3^*^	74.3 ± 2.2^**^	2.5 ± 0.2^*^
Sil	38.8 ± 0.8^*^	7. 9 ± 0.5^*^	70.5 ± 3.2^**^	3.6 ± 0.3^***^
CtN	63.4 ± 1.4	13.5 ± 0.5	115.1 ± 4.5	4.9 ± 0.3

Each value represents the mean ± SE of six rats. Ctrl: Control rats; Diab: Diabetic rats; CoN: Diabetic rats co-treated with naringenin during streptozotocin induction; PoN: Diabetic rats treated with naringenin after diabetes induction; Glib: Diabetic rats treated with standard drug glybenclamide; Sil: Diabetic rats treated with Silymarin; CtN: Control rats treated with naringenin. ^#^ Denotes significant difference compared with control rats; ^*^P < 0.05; ^**^P < 0.01; ^***^P < 0.001 denotes significant difference compared with diabetic control.

#### 3.3.2 GPx Activity

The activity of Glutathione peroxidase (GPx), a key redox regulator, was found to decrease significantly by 52.75% (*P* < 0.001) in diabetic rats as compared to non-diabetic rats. Significant enhancement of 56.43% (*P* < 0.01) and 77.80% (*P* < 0.01) in GPx activity was found in diabetic rats co and post treated with naringenin. The response to naringenin treatment was comparable to standard drug, glybenclamide and known free radical quencher silymarin (Table 3).

#### 3.3.3 SOD Activity

Free radicals generated due to diabetes induction caused alterations in SOD activity, the first line of antioxidant defense. As summarized in Table 3, significant (*P* < 0.001) decrease of 57.35% in SOD activity was observed in the liver of diabetic rats as compared to non-diabetic rats. Co-treatment of naringenin significantly (*P* < 0.001) enhanced SOD activity by 84.6%, as compared to diabetic rats. Standard drug glybenclamide was able to significantly restore the activity by 69.4%.

#### 3.3.4 Glutathione redox ratio

A decrease in the redox ratio (GSH/GSSG) indicates an increase in the reductive stress and marks the onset of various vascular diseases and complications. The diabetic group showed significant (*P* < 0.001) decrease in the redox ratio indicating the generation of oxidative stress. Co- treatment of naringenin increased the redox ratio by 3.77-fold (*P* < 0.001), while in its post exposure, it increased to 3.72-fold (*P* < 0.001) which was comparable to the increase observed due to glybenclamide administration (Table 3).

#### 3.3.5 Lipid peroxidation in diabetic rats

**Fig. 2:**
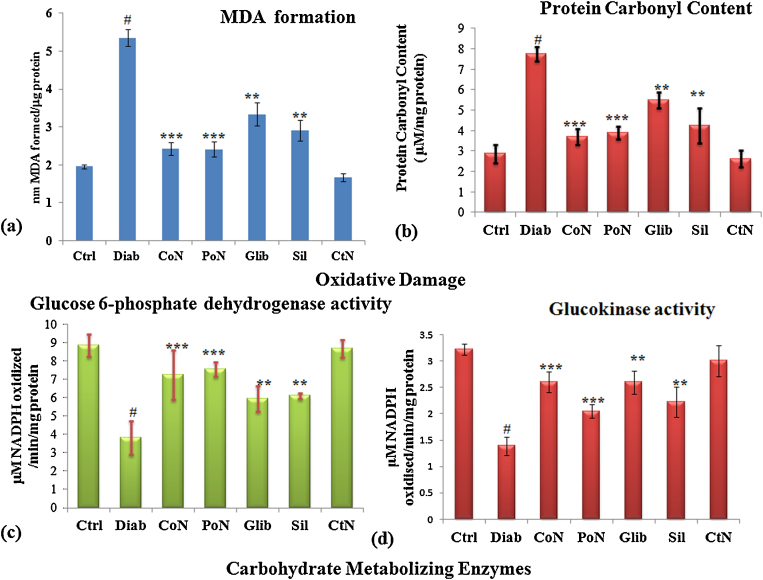
Effect of naringenin on oxidative damage and glucose metabolizing enzymes. (a) MDA levels in liver of control, diabetic and diabetic treated rats. (b) Protein carbonyl content in control, diabetic and diabetic treated rats. (c) Glucose-6 phosphate dehydrogenase activity in control, diabetic and treated rats. (d) Glucokinase activity in control, diabetic and treated diabetic rats. Each value represents the mean ± SE of six rats. # Denotes significant difference compared with control rats. *P < 0.05, **P < 0.01 and ***P < 0.001 denotes significant difference compared with diabetic control.

Free radicals generated due to hyperglycemia caused peroxidative damage to the tissues, leading to increase in levels of MDA (malondialdehyde) formation. The concentration of malondialdehyde, a terminal compound of lipid peroxidation, is commonly used as an index of oxidative injury due to free radicals In the liver of diabetic rats, significant (*P* < 0.001) increase of 2.74-fold in lipid peroxidation was found. A decrease of 2.21-fold (*P* < 0.001) in MDA formation was observed in diabetic rats co-treated with 50 mg/kg bwt naringenin. The decrease in MDA formation by naringenin during co- and post-treatment was similar to standard drug, glybenclamide (Fig. 2a).

#### 3.3.6 Protein carbonyl content

In the liver of diabetic rats, a significant increase in protein oxidation (2.71-fold) was seen as compared to the vehicle control group (Fig. 2b). However, Naringenin significantly decreased the protein oxidation level by 2.08-fold and 2.11-fold in co- and post-treatment, respectively (*P* < 0.001) compared with the non-treated diabetic rats. The response of naringenin was better as compared to standard drug glybenclamide. No significant damage was observed in control rats treated with naringenin.

### 3.4 Carbohydrate metabolizing enzymes

#### 3.4.1 Glucokinase

Glucokinase (EC 2.7.1.2), is an important glucose metabolizing enzyme, which facilitates glucose phosphorylation to glucose-6-phosphate. Glucokinase plays an important role in the regulation of carbohydrate metabolism by acting as a glucose sensor, and manages the glucose metabolism during its depletion or excess in blood. Significant (*P* < 0.001) decrease of 2.32-fold in hepatic glucokinase activity was seen in diabetic rats. Maximum enhancement of 1.88-fold in the activity of glucokinase was seen during co-treatment of naringenin (Fig. 2c).

#### 3.4.2 Glucose-6-phosphate dehydrogenase

During diabetes, change in glycolytic, NADPH generating and gluconeogenic enzymes is observed in liver. In the liver of diabetic rats, the level of glucose-6-phosphate dehydrogenase was significantly (*P* < 0.01) reduced by 2.31-fold, which was found to increase during different treatment schedules of naringenin. A maximum enhancement in the activity of glucose-6-phosphate dehydrogenase observed was 1.97-fold in co-treatment of naringenin (Fig. 2d) and the response due to phytochemical was comparable to glybenclamide.

### 3.5 ROS generation

**Fig. 3:**
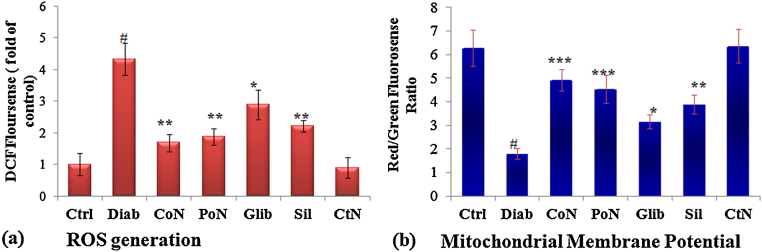
Protection accorded by naringenin in ROS generation and MMP of diabetic rats. (a) ROS generation assessed by spectrofluorometry using fluoroprobe DCFH-DA. Results are represented as fold of DCF fluorescence as compared to control. (b) Induction of mitochondrial membrane collapse in hepatocytes isolated from control, diabetic and diabetic treated rats. Mitochondrial membrane potential of treated and control cells was assessed by fluorescent spectrophotometer using JC-1. Decrease in the red (polarized)/green (depolarized) fluorescence ratio reflects increased number of depolarized mitochondria. Each value represents the mean ± SE of six rats. # Denotes significant difference compared with control rats. *P < 0.05, **P < 0.01 and ***P < 0.001 denotes significant difference compared with diabetic control. (For interpretation of the references to color in this figure legend, the reader is referred to the web version of the article.)

Various metabolic pathways are considered to cause an increase in ROS generation, during diabetes. Intracellular ROS generation in different treatment groups was estimated using DCFH-DA flouro-probe. Hepatocytes isolated from diabetic rats exhibited significantly enhanced ROS generation which was 4.33-fold (*P* < 0.001) as compared to control. Hepatocytes isolated from diabetic rats co- and post-treated with naringenin showed a significant (*P* < 0.001) decline in the ROS generation as assessed by DCFH flourescence, which was 2.57 and 2.34-fold decrease respectively in comparison to hepatocytes isolated from diabetic rats. Whereas, 1.60-fold decrease in ROS generation was observed in the glybenclamide treated group (Fig. 3a).

### 3.6 Mitochondrial membrane potential

Activation of a cell death program or apoptosis is triggered by initiation of certain cascadic events, which include mitochondrial depolarization and the subsequent release of pro-apoptotic factors. Alteration in mitochondrial trans-membrane electrical potential was investigated in primary hepatocytes isolated from rats of different treatment groups. As mitochondria get depolarized, fluorescence of the JC-1 dye changes from red to green. An increase of monomeric JC-1 molecules (green fluorescence) due to a decrease of mitochondrial membrane potential occurred in hepatocytes isolated from diabetic rats. A 3.53-fold (*P* < 0.001) decrease in the ratio of red to green fluorescence was seen in hepatocytes of diabetic rats indicating depolarization. Hepatocytes isolated from diabetic rats co-treated with naringenin exhibited a significant enhancement in red/green fluorescence with a maximum increase of 2.75-fold (*P* < 0.001) observed in hepatocytes isolated from naringenin co-treated rats (Fig. 3b) indicating prevention of depolarization of mitochondria.

### 3.7 Expression of Bax and Bcl-2

**Fig. 4:**
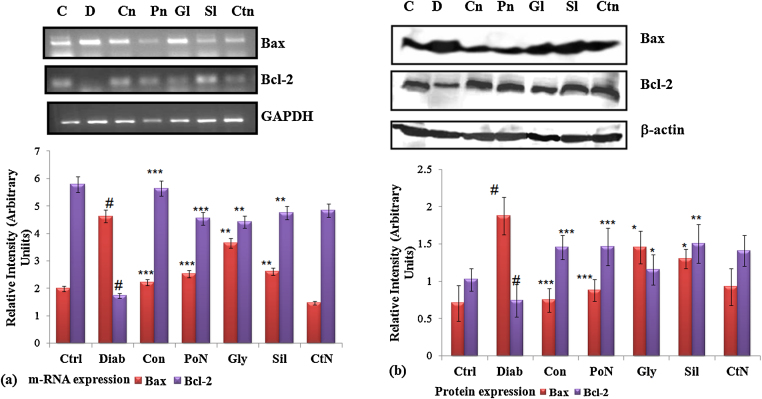
Effect of naringenin on transcriptional and translational level of Bax and Bcl-2 of control and diabetic rats. (a) Shows m-RNA level of Bcl-2 and Bax, using GAPDH as internal control. (b) Shows protein level of Bax and Bcl-2 as estimated through western blot, β-actin served as loading control. Densitometry shows relative intensity normalized to internal control. Results are shown as mean ± S.E. # Denotes significant difference compared with control values and *P < 0.05, **P < 0.01 and ***P < 0.001 denotes significant difference compared with diabetic rats. # Denotes significant difference compared with control values.

Depolarized mitochondria mark the release of various apoptotic and anti-apoptotic factors, which trigger the downstream cascade of cell death. We further investigated the scheme of events involved in diabetes-induced stress, which included changes in apoptotic genes like Bax, and antiapoptotic gene Bcl-2. Levels of apoptotic and anti-apoptotic genes and proteins were assessed. At the transcriptional level, the amount of Bax, which was increased by 2.33-fold (*P* < 0.001) in diabetic rats, showed a decline of 2.08-fold (*P* < 0.01) on treatment with naringenin. A significant increase of 2.70-fold (*P* < 0.001) in Bax protein level was detected in diabetic rats. Naringenin treated diabetic rats showed a corresponding decline (2.52-fold) in Bax protein level. Diabetes caused significant reduction in the level of Bcl-2, which was again restored by naringenin (3.62-fold). The effect of naringenin was much pronounced than that of standard drug glybenclamide and known antioxidant silymarin (Fig. 4).

### 3.8 Translocation of mitochondrial AIF and Endo-G

**Fig. 5:**
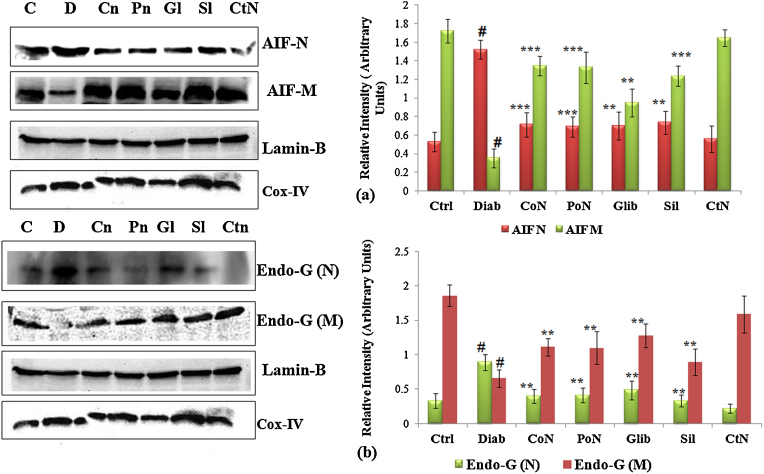
Translocation of AIF and Endo G from mitochondria to nucleus. Diabetes induced cell death involves AIF and Endo-G release from mitochondria. (a) Shows the changes in immunoreactivity for AIF in the mitochondrial and nuclear fractions. Mitochondrial AIF was depleted significantly after diabetes induction with Cox (IV) serving as internal control. (b) Shows Endo-G release from mitochondria due to permeability changes and its translocation to nucleus. Changes in the immuno-reactivity for Endo-G in the mitochondrial and nuclear fractions were assessed. Mitochondrial Endo-G was depleted significantly after diabetes induction. Cox (IV) served as internal control for mitochondria and Lamin-B served as internal control for nuclear fraction. Densitometry shows relative intensity normalized to internal control. Results are shown as mean ± S.E. # Denotes significant difference compared with control values and *P < 0.05, **P < 0.01 and ***P < 0.001 denotes significant difference compared with diabetic rats. # Denotes significant difference compared with control values.

During apoptosis, release of apoptotic proteins like AIF and Endo-G from mitochondria and then its translocation to the nucleus are considered important events. Release and translocation of these two important proteins under high glucose stress has been established in our earlier in vitro studies [14], [15]. During apoptosis, Endo-G which resides within the mitochondria translocates to the nucleus and causes large scale DNA fragmentation. To analyze the potential involvement of AIF and Endo-G during progression of diabetes and the onset of its complications, the protein levels of AIF and Endo-G were quantified by western blot in mitochondrial and nuclear fraction. In diabetic rats, a significant decrease of 4.91-fold in the level of AIF in mitochondria was accompanied by a substantial increase of 2.92-fold in the nuclear fraction. Treatment of diabetic rats with naringenin exhibited significant (*P* < 0.001) reduction of AIF in mitochondria. Similar to the pattern observed for AIF, diabetic rats showed a marked increase of 2.84-fold (*P* < 0.001) in the level of Endo-G in nuclear fraction and decrease in the mitochondrial fraction by 2.75-fold (*P* < 0.001). Level of translocated Endo-G was significantly modulated by naringenin. The protection accorded by naringenin was observed to be more pronounced than that of glybenclamide and silymarin (Fig. 5).

### 3.9 Study of caspase9/3 genes, proteins and caspase 3 enzymatic activity

**Fig. 6:**
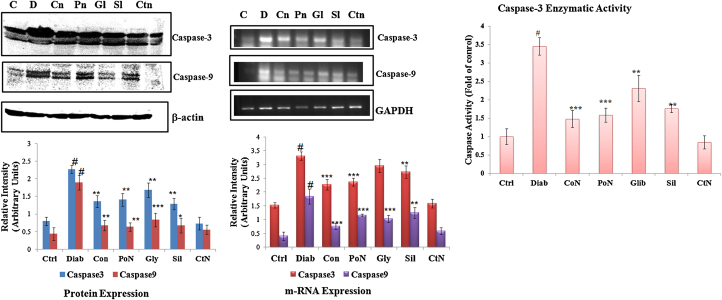
Effect of naringenin on transcript and protein levels of caspase-3/9 and enzymatic activity of caspase-3. (a) Shows activation of caspase-3 and caspase-9 as evident from the appearance of its cleaved product. (b) Shows m-RNA level of apoptotic genes-caspase-3 and caspase-9 using GAPDH as internal control. Densitometry shows the relative intensity normalized to internal control, (c) shows caspase-3 enzymatic activity of control and treated rats. Results are shown as mean ± S.E. # Denotes significant difference compared with control values and *P < 0.05, **P < 0.01 and ***P < 0.001 denotes significant difference compared with treated rats.

Release of various apoptotic factors like Bax, AIF and Endo-G result in increased expression level of effector and executor caspases. With this view, the expression levels of caspase-9 and caspase-3 were determined along with caspase-3 activity. Diabetic rats exhibited an increased gene expression of caspase-3 (*P* < 0.001) by 2.17-fold. Expression of caspase-3 was decreased in naringenin co-treated rats by 1.48-fold (Fig. 6). The result obtained from the gene expression study was consistent with results obtained in protein expression. Enzymatic activity of caspase-3 was significantly (*P* < 0.01) increased by 3.47-fold in diabetic rats. Naringenin modulated the activity and a significant reduction of 2.35-fold (*P* < 0.001) was observed with co-treatment of naringenin. A similar response was observed during post-treatment with naringenin and silymarin, which was more efficient than that of standard drug glybenclamide. The expression level of caspase-9 was reduced (*P* < 0.001) by 1.89-fold and was significantly ameliorated by naringenin at both transcriptional and translational level.

### 3.10 DNA fragmentation

**Fig. 7:**
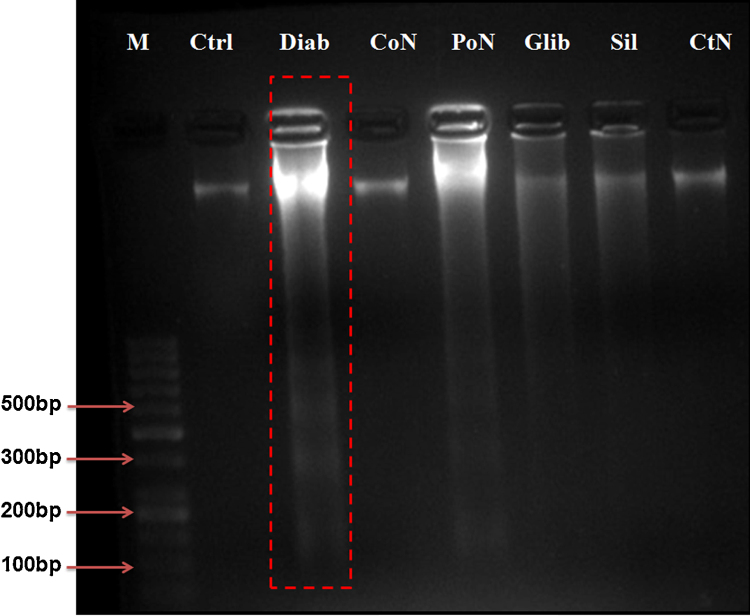
Modulation of diabetes induced DNA fragmentation. Intra-nucleosomal DNA fragmentation in control, diabetes, diabetes treated rats (a). Lane 1: DNA Marker 100 bp: Lane 2: DNA isolated from Control rats, Lane 3: DNA from diabetic rats Lane 4: Naringenin co-treated diabetic rats. Lane 5: Naringenin post-treated diabetic rats, Lane 6: Diabetic rats treated with Glybenclamide. Lane 7: Diabetic rats treated with Silymarin, Lane 8: Control rats treated with naringenin.

Certain morphological and biochemical characteristics are related to apoptotic cell death. One such qualitative feature of apoptosis is DNA fragmentation. In cells undergoing apoptosis, DNA is fragmented into smaller fragments of about 180-bp oligomers and multiple thereof (360, 540, etc.), which appears as a DNA ladder when run on an agarose gel. DNA samples from the control rats and rats post- and co- treated with naringenin showed no distinct bands, but high molecular weight was retained on the top portion of the gel (Fig. 7). In contrast, a number of low molecular weight bands showing specific laddering pattern were observed in the DNA samples obtained from diabetic rats. Control rats treated with naringenin did not show DNA fragmentation.

## 4 Discussion

Diabetes is a complex metabolic disorder with characteristic modulation of glucose metabolism leading to excessive ROS production and generation of various diabetic complications like nephropathy, neuropathy, cardiopathy and even hepatopathy. Various glucose metabolic pathways are mainly responsible for the generation of ROS and are involved in many of the micro- and macrovascular complications that are associated with diabetes. Under normal conditions, ROS at its low level functions as “redox messenger” in intracellular signaling and regulation. On the contrary, when major glucose metabolizing pathways come into action, ROS is generated leading to oxidative modification of cellular macromolecules, disturbed protein function and activated cell death signaling. High blood glucose, in addition to the generation of free radicals, depresses the level of natural antioxidant defense compounds such as ascorbic acid and GSH present in the body [30], thereby causing major antioxidant imbalance. Hence, management and sustenance of antioxidant status can be an effective panacea for diabetes. In our earlier studies, we have reported the relation of high glucose to the generation of ROS and onset of apoptosis in cultured primary hepatocytes. Based on our previous findings [14], [15], the present in vivo study was taken up to further investigate the molecular mechanisms underlying diabetes-induced apoptosis in liver of rats and its amelioration by natural phyto-constituent naringenin.

In the present investigation, we observed the anti-hyperglycaemic effect of naringenin through the oral glucose tolerance test in which even 50 mg/kg bwt dose significantly improve glucose tolerance. Induced diabetes is marked by severe diminution of body weight, which may be attributed to loss or degradation of structural proteins [41]. In our present study, we observed that diabetes-induced decrease in body weight was prevented by naringenin, which is in accordance with earlier studies reporting, prevention of body weight loss in diabetic rats by the usage of phytochemicals or medicinal plant extracts [30].

Glucokinase is one of the important enzymes in the glucose catabolism which phosphorylates glucose to glucose-6-phosphate [1]. In our study, we report decreased glucokinase activity in the liver of diabetic rats, as reported earlier [22], [25], [26], [30]. Diabetic rats treated with naringenin (50 mg/kg bwt) showed an enhancement in glucokinase activity to near control levels. We also found a decrease in the activity of glucose 6-phosphate dehydrogenase in diabetic rats. The literature suggests the decrease in this enzyme activity can inhibit the pentose phosphate pathway in diabetic conditions [1]. Diabetic rats treated with naringenin and silymarin significantly enhanced liver glucose-6-phosphate dehydrogenase activity. This results in an increased production of the reducing agent, NADPH, with the collateral decrease in oxidative stress.

Role of ROS in the generation and progression of diabetes is well postulated and has been reported by many researchers. Our findings on the disturbed antioxidant status due to diabetes are in accordance with various earlier reports [13], [16], [43]. Diabetes-induced free radicals caused peroxidative damage to the tissues, leading to increase in the levels of MDA formation and protein carbonyl content. Free radicals generated due to different glucose metabolizing pathways can be well abrogated by phytochemicals having good antioxidant property. Naringenin, a flavonoid, was able to restore the modulation seen in SOD, Catalase, GPx activity and redox ratio due to diabetes induction. Moreover, naringenin showed significant reduction of lipid peroxidation and protein carbonyl content in diabetic rats. These results support the earlier findings reporting the use of phytochemicals in effective treatment of tissue damage during diabetes [22], [43]. The in vivo hepatoprotection and antioxidant effect of naringenin could be attributed to its anti-hyperglycaemic effect. Authors hence presume that by preventing hyperglycaemia the diabetes-induced liver damage may be prevented.

Mitochondrial dysfunction has been proposed as a critical modulator of ROS generation and onset of apoptosis during diabetic complications. Loss of mitochondrial membrane potential is observed due to excess mitochondrial ROS production. Depolarization of the mitochondrial inner membrane potential (Δψm) in streptozotocin-induced diabetic rats suggests the loss of mitochondrial membrane integrity and formation of mitochondrial pore. Several studies using in vitro cell culture or in vivo animal models, including our own, have suggested that high glucose cause loss of mitochondrial membrane potential [13], [14], [15], [38], [43]. Several apoptogenic proteins like Cyt-c, AIF, and Endo-G are said to be released through pores formed in mitochondrial membrane. Naringenin, by preventing excessive ROS production, was effective in maintaining the mitochondrial membrane potential and thereby preventing the release of various apoptogenic factors from mitochondria. As a consequence, the expression of caspase-3 and caspase-9 which was upregulated in diabetic rats due to release of apoptogenic factors from mitochondrial pores was prevented in naringenin treated rats, thus checking the formation of apoptosome with caspases.

An important finding in this study is that diabetes causes the release of certain factors like AIF and Endo-G that play an important role in DNA damage. With the formation of pore in the mitochondrial membrane, factors like AIF and Endo-G leak out and cause integral damage to DNA and nuclear material. Streptozotocin induced diabetic rats showed release of these proteins, suggesting their translocation from mitochondria to the nucleus is an important step required for apoptosis to take place. Our findings regarding the release of these apoptogenic factors are in accordance with earlier studies reporting the release of AIF and Endo-G during diabetes [8], [9]. We have also reported the involvement of these two proteins in the development of apoptosis under high glucose stress [14], [15]. With the translocation of AIF and Endo-G from its resident place, the enhanced DNA damage was seen in diabetic rats. Naringenin, by restoring mitochondrial membrane potential, restrained the translocation of AIF and Endo-G to the nucleus which precluded DNA damage and thereby prevented tissue damage associated with diabetes. Inhibition of apoptosis or redox imbalance was more pronounced in the case of naringenin as compared to standard drug glybenclamide or known plant-derived antioxidant silymarin. This property of naringenin marks it as a potential candidate for management of diabetes under the experimental conditions.

## 5 Conclusion

In the present study, various events related to generation and progression of diabetic complications during streptozotocin induced diabetes was investigated. The current findings propose sequential events that lead to diabetic hepatopathy. Decreased body weight, altered activity of liver and kidney marker enzymes, altered carbohydrate metabolizing enzyme activities, analyses of the expression of Bcl-2 family genes and proteins, antioxidant enzyme activities, tissue MDA formation, generation of ROS, translocation of AIF, Endo G and activation of caspase 9/3 suggest that apoptosis involve in diabetic hepatopathy is via activation of the AIF-caspase independent/mitochondrial apoptotic pathway and DNA damage. Naringenin, an active constituent of traditionally used antidiabetic plant *Citrus paridisi*, exerted the anti-hyperglycemic effect by preventing oxidative stress and resultant apoptotic events during diabetes-induced liver damage. Taken together, these findings suggest that naringenin has potential as adjunct therapy for the management of diabetic hepatopathy

## Conflicts of interest

None declared.

## Transparency document

Transparency document
