# Supplementary material for: Naringenin accords hepatoprotection from streptozotocin induced diabetes in vivo by modulating mitochondrial dysfunction and apoptotic signaling cascade
Source: Toxicol Rep. 2014 Aug 13;1:569–81. doi: 10.1016/j.toxrep.2014.08.002 (PMC5598533; doi:10.1016/j.toxrep.2014.08.002)
Supplement: Supplementary file 2 [file mmc2.doc]

**Naringenin accords hepatoprotection from streptozotocin induced diabetes in vivo by modulating mitochondrial dysfunction and apoptotic signaling cascade**

**Radhika Kapoor and Poonam Kakkar**

CSIR-Indian Institute of Toxicology Research, Lucknow, India

*Corresponding Author

Dr. Poonam Kakkar

Chief Scientist & Head

Herbal Research Section

Food Drug and Chemical Toxicology Division

CSIR-Indian Institute of Toxicology Research

P.O. Box-80, M.G.Marg

Lucknow-226001

Email: [poonam_kakkar@yahoo.com; pkakkar@iitr.res.in](mailto:poonam_kakkar@yahoo.com; pkakkar@iitr.res.in)

Tel: +91-522-2627586/2613786 Extn. 269

Fax: +91-522-2628227

**Material and methods**

1. ***Measurement of body weight.***

Body weight of all groups of animals was monitored on a daily basis at a fixed time. Fixed amount of rat chow and fluid was given to each rat and replenished the next day.

**Results:**

**Effect of naringenin on body weight of treated and un-treated diabetic rats.**

Though initial weight of all groups was almost similar, diabetes caused a significant reduction (P<0.001) in body weight. Naringeinin during its co-treatment prevented the decrease in body weight caused due to diabetes. Significant increase of 2.08 fold in body weight of diabetic rats was observed when co-treated with naringenin as compared to untreated diabetic rats. No significant change in weight of control rats treated with naringenin was observed (supplementary data Fig S1).


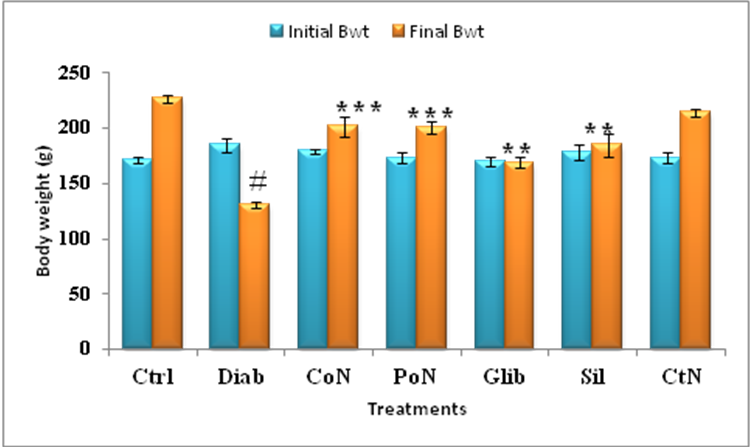


**Fig S1: Body weight of treated and untreated diabetic rats.**

Each value represents the mean ± SE of six rats. # denotes significant difference compared with control rats. *P < 0.05, **P < 0.01 and ***P < 0.001 denotes significant difference compared with diabetic control. Ctrl: Control rats; Diab: Diabetic rats; CoN: Diabetic rats co-treated with naringenin during streptozotocin induction; PoN: Diabetic rats treated with naringenin after diabetes induction; Glib: Diabetic rats treated with standard drug glybenclamide; Sil: Diabetic rats treated with Silymarin; CtN: Control rats treated with naringenin
